# A Large-Size MEMS Scanning Mirror for Speckle Reduction Application †

**DOI:** 10.3390/mi8050140

**Published:** 2017-05-03

**Authors:** Fanya Li, Peng Zhou, Tingting Wang, Jiahui He, Huijun Yu, Wenjiang Shen

**Affiliations:** 1 The School of Materials Science and Engineering, Xi’an Jiaotong University, Xi’an 710049, China; fyli2016@sinano.ac.cn (F.L.); ttwang2014@sinano.ac.cn (T.W.); jhhe2014@sinano.ac.cn (J.H.); 2 Key Lab of Nanodevices and Applications, Suzhou Institute of Nano-tech and Nano-bionics, Chinese Academy of Sciences, Suzhou 215123, China; pzhou2015@sinano.ac.cn (P.Z.); hjyu2012@sinano.ac.cn (H.Y.)

**Keywords:** microelectronic mechanical system (MEMS), speckle reduction, electromagnetic force, optical scanning

## Abstract

Based on microelectronic mechanical system (MEMS) processing, a large-size 2-D scanning mirror (6.5 mm in diameter) driven by electromagnetic force was designed and implemented in this paper. We fabricated the micromirror with a silicon wafer and selectively electroplated Ni film on the back of the mirror. The nickel film was magnetized in the magnetic field produced by external current coils, and created the force to drive the mirror’s angular deflection. This electromagnetically actuated micromirror effectively eliminates the ohmic heat and power loss on the mirror plate, which always occurs in the other types of electromagnetic micromirrors with the coil on the mirror plate. The resonant frequency for the scanning mirror is 674 Hz along the slow axis, and 1870 Hz along the fast axis. Furthermore, the scanning angles could achieve ±4.5° for the slow axis with 13.2 mW power consumption, and ±7.6° for the fast axis with 43.3 mW power consumption. The application of the MEMS mirror to a laser display system effectively reduces the laser speckle. With 2-D scanning of the MEMS mirror, the speckle contrast can be reduced from 18.19% to 4.58%. We demonstrated that the image quality of a laser display system could be greatly improved by the MEMS mirror.

## 1. Introduction

Solid-state lasers can provide wider color gamut, longer lifetime, and higher brightness and contrast of images compared to light emitting diodes (LEDs), a popular light source for projection displays [1]. Laser display technology plays a significant role in our life, and can be applied in various fields such as movie theatres, home televisions and conference rooms. However, the existence of speckle degrades the images quality severely, which is an irregularly distributed pattern of light and dark particles caused by the interference of the reflective coherent laser beam from the rough screen comparable to optical wavelength [2,3,4]. One of the promising speckle reduction technologies in laser projection [5,6] is to employ MEMS scanning mirrors. At present, research on MEMS scanning mirrors are mostly focused on small diameter MEMS mirrors, while rarely on the larger size. Large-size mirrors can not only tolerate high optical power, but also ensure maximum utilization of light energy [7]. Microvision Company in the United States developed an electromagnetic two-dimensional scanning mirror, and successfully applied it to a laser Pico projection system, but the 1-mm diameter of the mirror was unable to meet the requirements of the high lumen imaging display [8]. Oliveira et al. were the pioneers who applied a MEMS scanning mirror to eliminate laser speckle. Limited to a 0.8-mm diameter, the MEMS mirror had issues when used in practical laser display systems [9]. Akram et al. in Vestfold University in Norway further improved the MEMS two-dimensional scanning mirror and improved the quality of the laser display images, but its diameter was only 2 mm, and also cannot be used in high power laser displays [10]. The large size and mass of a MEMS two-dimensional mirror limit the possibility of achieving a larger angle unless the driving moment is high enough. At the same time, the oscillating micromirror used to reduce the laser speckle should also have a high operating frequency [11].

Based on the above requirements, this paper proposes a 6.5-mm diameter, two-dimensional MEMS scanning mirror driven by the electromagnetic method. The efficient electromagnetic drive mode not only offered the driving moment of the large angle required, but also realized the high frequency. The mirror is used in a laser projection system to suppress laser speckle. This scanning mirror with a large diameter could be used in high power laser illumination for high lumen projection. Moreover, the high frequency of the scanning mirror could effectively reduce the speckle contrast and bring clearer and more comfortable images.

## 2. Design

**Figure 1:**
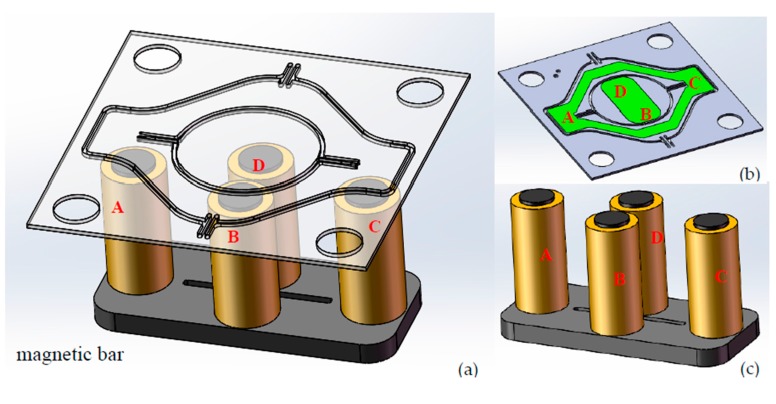
(**a**) Illustration of the microelectronic mechanical system (MEMS) mirror and the actuation coils underneath; (**b**) Back profile of mirror with nickel; (**c**) External coils.

This study employed the electromagnetic scanner in Figure 1 to demonstrate the proposed design concept. As indicated in Figure 1a, four external coils (A, B, C, and D) are symmetrically placed in corresponding positions and kept a certain distance for the mirror’s free deflection. Specifically, the coils A and C are located beneath the ferromagnetic film on the outer frame, and coils B and D are placed beneath the rectangular ferromagnetic film on the back of the mirror. Coils A and C are responsible for the slow axis, while B and D coils are for the fast axis. When the A and C coils are driven by the square wave signals with the same frequency and 180° phase difference, the two coils, the bottom magnetic bar and the soft Ni film on the outer frame will compose a closed magnetic circuit, then an attractive force will drive the mirror to deflect a certain angle around the slow axis. Similarly, coils B, D, the bottom magnetic bar and the soft Ni film will compose another magnetic circuit when excitation signals are applied to B and D coils, so as to achieve the purpose of the two-dimensional scanning with our proposed model. Figure 1b,c shows the backside of mirror and the external coils, respectively. The two groups of coils and the signals are controlled separately, therefore, good independence, scanning linearity and accuracy can be achieved for the biaxial scanner. Detailed dimensions of the scanner and coils in our design are summarized in Table 1.

**Figure 2:**
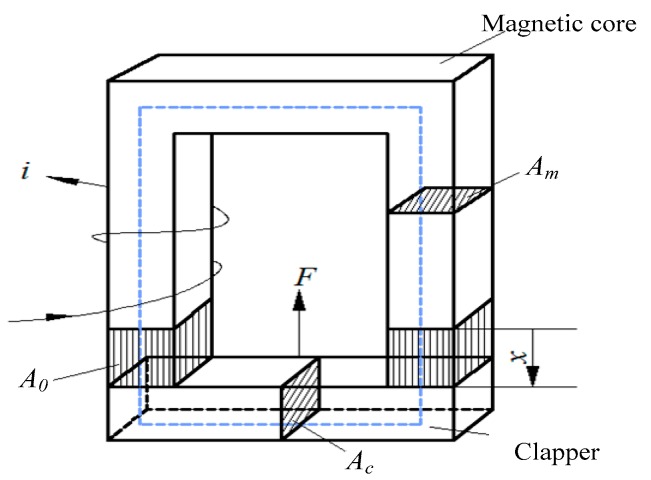
A magnetic circuit model consists of a magnetic core with copper winding and a clapper made out of ferromagnetic materials. The magnetic field created by the copper coil is concentrated in the magnetic core and clapper due to their high permeability. The magnetic circuit path is shown by dashed lines.

We introduced a magnetic circuit model to solve the theoretical value of force shown in Figure 2. Based on the hypothesis [12] that all the magnetic fluxes pass through the core (no leakage except for the air gap), and according to the Maxwell’s magnetic force formula, attractive force could be expressed by:

(1) $$F\;=\;\frac{B_{0}{}^{2}A_{0}}{\mu_{0}}$$

In the equation, $B_{0}$ is defined as the magnetic flux density of the air gap, $A_{0}$ is the cross-sectional area of the gap, and $\mu_{0}$ is the magnetic permeability of air. According to the Ampere’s Law, we could derive that:

(2) $$N\cdot i\;=\;\oint^{\text{​}}{Hds\;=\;H}_{m}l_{m}{+\;H}_{c}l_{c}{\;+\;H}_{0}\cdot 2x\;,$$

where N is the number of coil turns, and i is the current flowing through the coil. $H_{m}$, $\;H_{c}$,$\;\mathrm{and}\;H_{0\;}$ respectively represent the strength of the magnetic field for the magnetic core, the clapper (ferromagnetic film) and the gap. $l_{m}$, $l_{c}$, and x represent the length of the magnetic core, the clapper and the air gap.

Because:

(3) $$H\;=\;\frac{B}{\mu }\;=\;\frac{\Phi }{\mu \;A}$$

the permeability of air can be negligible compared with the ferromagnetic materials’ permeability, that is to say:

$$\mu_{0}\ll \mu_{m},\;\mu_{0}\ll \mu_{c}$$

By assuming:

(4) $${\;A}_{c\;}{=\;A}_{m}{=\;A}_{0\;}=\;A$$

Then, substituting (2)–(4) into the Equation (1), the magnetic force can be expressed as follows:

(5) $$F\;=\;k\frac{i^{2}}{x^{2}}$$

where $k\;=\;\frac{\mu_{0}N^{2}A}{4}$. Obviously, the value of the driving force is proportional to the square of the current and inversely proportional to the square of the gap length.

## 3. Fabrication

**Figure 3:**
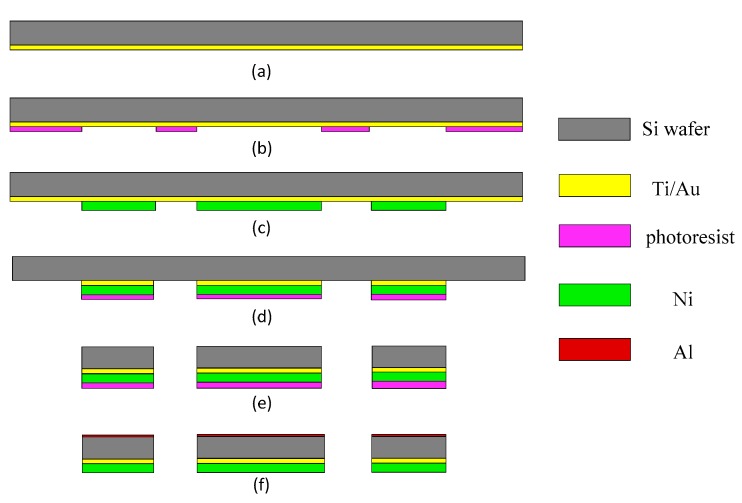
Fabrication flow for the MEMS mirror. (**a**) Ti/Au seed layer sputtering; (**b**) thick photoresist (AZ4620) spinning and exposure; (**c**) nickel electroplating and photoresist removal; (**d**) second photolithography and Ti/Au films removal; (**e**) Si etching (DRIE); (**f**) Al layer coating.

Figure 3 shows the detailed fabrication process. As in Figure 3a, we used a double sided polished *n*-type (100) 200-μm thick Si wafer as the starting substrate. A 20-nm Ti adhesion layer and a 100-nm Au coating were sputtered on the backside of Si substrate as the seed layer for the following electroplating step. A 20-μm thick film of photoresist (AZ4620) was patterned to selectively electroplate nickel. After the soft-magnetic Ni electroplating step, the photoresist and the Ti/Au thin films were removed by acetone solution and IBE (ion beam etching) technology, respectively. Then, a second photolithography step was used to define the window for bulk silicon etching, as shown in Figure 3d. After that, the structure of the mirror plate and axes were released by the deep reactive ion etching (DRIE) process. Lastly, the front side of the silicon was coated with a 120-nm aluminum layer to form the reflective mirror surface.

## 4. Characterization and Results

**Figure 4:**
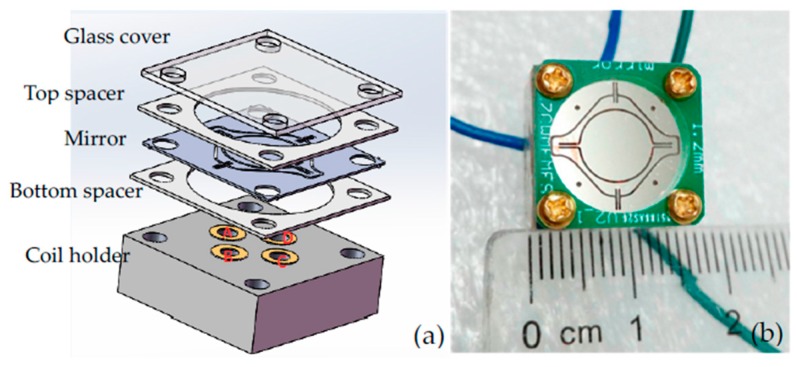
(**a**) Package method; (**b**) Fully assembled prototype.

Figure 4 shows the package method and fully assembled prototype. The whole package is similar to the sandwich structure: The uppermost glass cover is to protect the mirror from the external environment; a 1.2-mm thick plastic top spacer above the mirror is chosen to ensure a large incident angle; the distance between the Ni film and the bottom coils is about 0.45 mm, which is the thickness of the bottom spacer. The thickness of the bottom spacer defines the maximum allowable rotation angle; coils are symmetrically placed in the coil holder, and the driving currents are applied through electrical connections to the printed circuit board (PCB) pads. The whole device size is only 16 mm × 16 mm × 12 mm.

**Figure 5:**
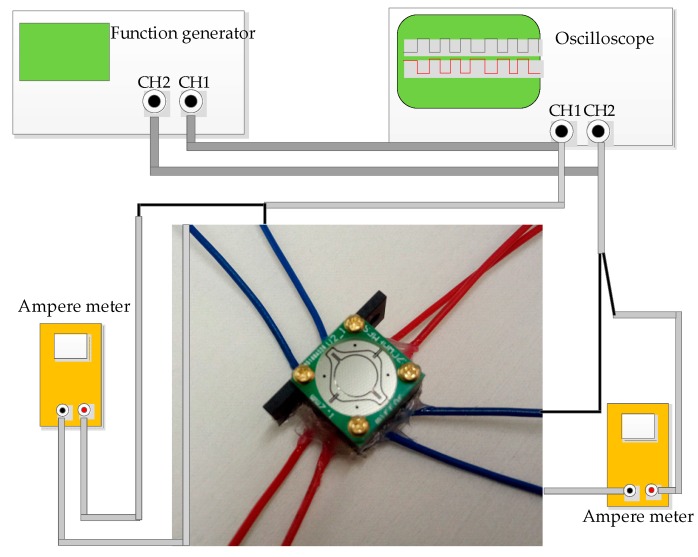
Schematic measurement setup: the 2-D scanning mirror was driven independently by function generators.

After packaging, the device was tested with the setup shown in Figure 5. In the measurement, two Ampere meters were used to record the relation between the current and the mirror’s scanning property. A function generator was employed to send square waves with a certain frequency to the coils as the excited signal, and to the oscilloscope for monitoring phase difference between input channels. The 2-D scanning mirror’s vertical and horizontal axes were driven independently by signals from the function generator.

**Figure 6:**
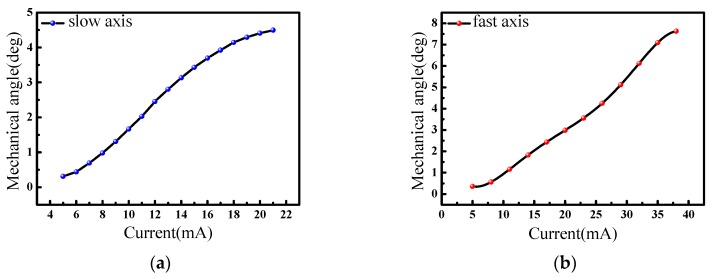
Current-angle relationship: (**a**) slow axis; (**b**) fast axis.

**Figure 7:**
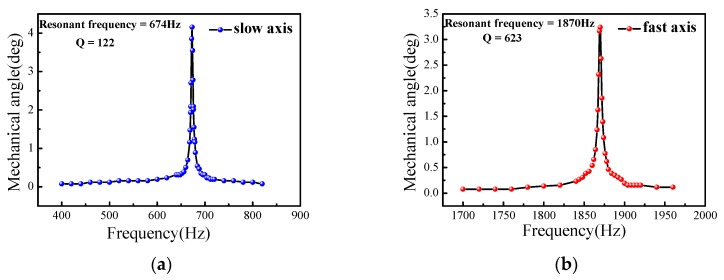
Frequency response: (**a**) slow axis; (**b**) fast axis.

The results are plotted in Figure 6. The deflection angle can be calculated from the length of the scanning line and the distance between the screen and the scanning mirror. When the micro-mirror worked at the resonant vibration state, mechanical torsion angles increased with the AC (alternating current) driving currents (refer to root-mean-square values of the current flowing in the coils) for both the slow axis and fast axis. The slow scanning angle could achieve ±4.5° at the applied current of 21 mA and power consumption of 13.2 mW, and the fast scanning angle could reach ±7.6° at the applied current 38 mA and maximum power consumption of 43.3 mW. We used a 0.45-mm thick bottom spacer here, so the maximum allowable rotation angle for the slow axis and fast axis were ±4.5° and ±8° theoretically, matching well with the experimental data. By tuning the driving frequency, the frequency response of the scanning angle could be recorded. Figure 7 shows the frequency responses of the slow and fast axes when 20 mA was applied to a coil. According to the results, the resonant frequencies were 674 Hz and 1870 Hz for the slow and fast axes, respectively. The quality factor *Q* was calculated by the following equation [13]:

(6) $$Q\;=\;\frac{f_{0}}{\Delta f}$$

where $\;f_{0}$ is the resonant frequency, and Δf is the half-power bandwidth. From the measured curve, we can derive the *Q* value; 122 for the slow axis and 623 for the fast axis. The large difference of *Q* values between the slow and fast axes was due to the dependence of damping on the resonant frequency. The *Q* value increases with the resonant mode and is proportional to f^0.5^ according to Chu et al. [14]. As shown in Figure 1, the slow scanning of the mirror is driven by *A* and *C* coils, and the outer frame is scanning together with the mirror plate. So, the air damping is severer and the *Q* value is smaller for the slow axis scanning.

Reliability is crucial to the successful application of MEMS devices when they reach commercialization [15,16]. To study the shock resistance of our fabricated devices, a shock test was performed in air at room temperature as follows [17]. A scanner prototype was fixed to a shock table by 3 M Epoxy Adhesive. Acceleration corresponded to the height of the table from which it was dropped. A piezoelectric transducer (PZT) sensor was mounted to the shock table to record actual acceleration. In the test, we changed the fixed direction so that the shock was applied in three (X, Y, Z) orientations, where the X and Y directions were along the fast axis and the slow axis respectively, and the Z direction was perpendicular to the mirror plane. The table was dropped to the floor three times in a row with three orthorhombic orientations, and half sine shock pulses with certain widths were produced. No significant fracture or electrical failures were observed until the prototype was tested at 900 g for X direction and 1500 g for Y, Z directions, where g is the gravity acceleration (g ≈ 9.8 m/s^2^), demonstrating that the micro-mirror and applied package structure have good shock resistance. In addition, a vibration test was carried out on Electro Dynamic Shakers with varying frequencies (from 20 Hz to 2000 Hz) at a constant acceleration of 20 g [17]. After three periods of shaking, the device could still operate as before. These results show that the device is reliable and durable for practical applications.

## 5. Application to Speckle Reduction

In the application to laser projectors, the speckle phenomenon emerges by reflecting highly coherent laser beams with single wavelengths on random rough surfaces, resulting in a random spatial intensity distribution [18]. One of the criterion to describe speckle is speckle contrast ratio, which is defined as [19]:

(7) $$C\;=\;\frac{\sqrt{\langle I^{2}\rangle {-\langle I\rangle }^{2}}}{\left\langle I\right\rangle }\times 100\%$$

where $\langle I^{2}\rangle \;$ and ⟨I⟩  represent the square mean value and the mean light intensity,$\;\sqrt{\langle I^{2}\rangle -{\left\langle I\right\rangle }^{2}}$ denotes the standard deviation. The lower the *C* value, the clearer images could be derived, which is now of great concern. 

**Figure 8:**
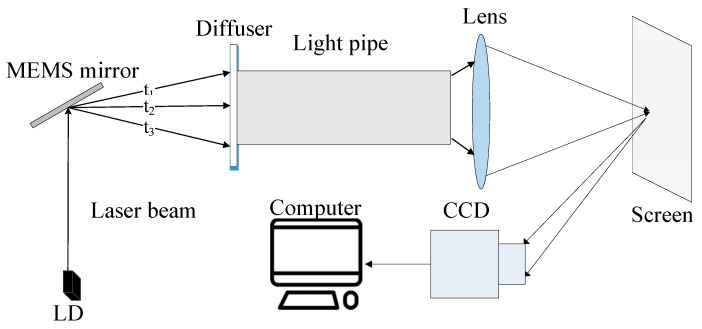
Speckle reduction system.

As presented in Figure 8, the simplified speckle reduction system consists of a laser diode, a fabricated scanning mirror, a light pipe, optics elements such as a focusing lens and a diffuser with high transmittance, and a charge-coupled device (CCD) camera for acquiring pattern information. With the two-dimensional scanning of the mirror, the laser beam was reflected onto the diffuser placed at the entrance of the light pipe with angle diversity at different times. After multiple reflections inside the light pipe, the uniform illumination will be formed at the exit surface, which, with the existence of subsequent imaging optics, form the picture on the screen. The scanning area can be changed with different driving currents, however, we need to control the total reflected light entering into the light pipe. According to speckle suppression theory [20,21], once the speckle images at different times and different positions are uncorrelated, then these irrelevant speckle figures are finally superimposed on each other during a frame image formed on the screen. If *N* independent speckle patterns are overlapped on an intensity basis, and we assume that each pattern has an equal mean intensity, the speckle contrast *C* in the integrated image is reduced to [4]:

(8) $$C=\;\frac{1}{\sqrt{N}}$$

where *N* is the number of independent speckle patterns. We could derive that the higher the value of *N*, the lower the value of speckle contrast results.

**Figure 9:**
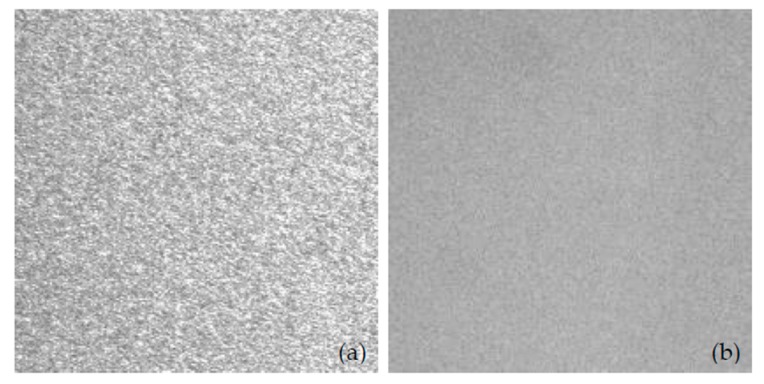
Speckle contrast images when CCD integration time was set to 50 ms: (**a**) Speckle pattern with inactive scanning mirror, *C* = 18.19%; (**b**) Speckle reduction pattern with active mirror, *C* = 4.58%.

In our measurement, the focus length and aperture f-number of the CCD imaging lens are 25 mm and 8, respectively. The CCD has a pixel size of 3.75 μm × 3.75 μm and is located at 2 m away from the screen. We explored the mirror’s stationary and vibrating conditions and their influence on the speckle contrast ratio. The calculated data by Matlab based on Equation (7) is presented in Table 2. When the mirror was working, the contrast was 4.58% during 50 ms integration time of the CCD camera, and when the mirror was turned off, the contrast was 18.19% at the same integration time. Figure 9 shows the speckle contrast images with and without the working mirror. With the 2-D scanning of the MEMS mirror, the speckle contrast for the laser projection display could be reduced from 18.19% to 4.58%. This result demonstrated that the scanning of the mirror can disturb the spatial and temporal coherences of the laser source and suppress the speckle pattern for laser projection images.

## 6. Conclusions

We have proposed and fabricated a large-size MEMS scanning mirror. Our mirror can meet the requirements of high resonant frequency and large deflection angle used for speckle reduction application, and successfully reduces the laser speckle contrast from 18.19% to 4.58%. In addition, the fabricated devices have a shock resistance of more than 900 g and good vibration resistance, which is also crucial when used in commercial applications.

## Figures and Tables

**Table 1:** The dimensions of designed scanner and coils.

Parameter		Value	Unites
Diameter of the mirror		6.5	mm
Thickness of the mirror		200	μm
Width of the axis	Slow axis	100	μm
	Fast axis	160	
Length of the axis	Slow axis	1500	μm
	Fast axis	1750	
Thickness of the axes		200	μm
Thickness of the nickel film		20	μm
Outer diameter of the coil		3	mm
Inner diameter of the coil		2	mm
Height of the coil		10	mm
Number of turns for the coil		900	-
Resistence of the coil		30	Ω

**Table 2:** The speckle contrast by different measurement systems.

Measurement System	Integration Time/ms	Maxmium Intensity	Minimum Intensity	Mean Intensity	Contrast Value/%
With mirror	50	157	108	126	4.58
Without mirror	50	255	94	179	18.19
